# From phenotypic to molecular diagnosis: Insights from a clinical immunology service focused on inborn errors of immunity in Colombia

**DOI:** 10.7705/biomedica.7533

**Published:** 2024-12-23

**Authors:** Mónica Fernandes-Pineda, Andrés F. Zea-Vera

**Affiliations:** 1 Departamento de Medicina Interna, Universidad del Valle, Cali, Colombia Universidad del Valle Departamento de Medicina Interna Universidad del Valle Cali Colombia; 2 Departamento de Microbiología, Facultad de Salud, Universidad del Valle, Cali, Colombia Universidad del Valle Departamento de Microbiología Facultad de Salud Universidad del Valle Cali Colombia; 3 Genetic Immunotherapy Section, Laboratory of Clinical Immunology and Microbiology, National Institute of Allergy and Infectious Diseases, National Institutes of Health, Bethesda, Maryland, USA National Institutes of Health National Institutes of Health Bethesda Maryland USA

**Keywords:** Molecular diagnostic techniques, autoimmunity, primary immunodeficiency diseases., técnicas de diagnóstico molecular, autoinmunidad, enfermedades de inmunodeficiencia primaria.

## Abstract

**Introduction.:**

Inborn errors of immunity include a broad spectrum of genetic diseases, in which a specific gene mutation might alter the entire emphasis and approach for an individual patient.

**Objective.:**

To conduct a comprehensive analysis of the correlation between phenotypic and molecular diagnoses in patients with confirmed inborn errors of immunity at a tertiary hospital in Cali, Colombia.

**Materials and methods.:**

We conducted a retrospective study in which we sequentially evaluated all available institutional medical records with a diagnosis of inborn errors of immunity.

**Results.:**

In the Clinical Immunology Service of the Hospital *Universitario del Valle*, 517 patients were evaluated. According to the IUIS-2022 classification, 92 patients (17.35%) were definitively diagnosed with an inborn error of immunity. Of these, 38 patients underwent genetic studies. The most prevalent category was predominantly antibody deficiencies (group III) (38/92 - 41.3%). A broad spectrum of genetic defects, novel and previously reported, were described, including mutations in the following genes: *ATM, BTK, ERBIN, MAB21L2, RAG2, SAVI, SH2D1A, STAT1, SYK*, and *TMEM173*. Less frequent findings included cases of the WHIM syndrome, SYKgain-of-function, and IL-7 deficiency.

**Conclusions.:**

The establishment of the Clinical Immunology Service in the *Hospital Universitario del Valle* has emerged as a pivotal resource, catering to individuals with limited financial means and covered by public health insurance within the southwest region of Colombia. Molecular genetics confirmatory diagnosis was achieved in 38 patients (41.3%) with inborn errors of immunity and changed the diagnosis in 24 cases (26%).

## Resumen

**Introducción.:**

Los errores innatos de la inmunidad incluyen un amplio espectro de enfermedades genéticas, en las cuales una mutación puede alterar completamente el enfoque y tratamiento de cada paciente.

**Objetivo.:**

Hacer un análisis exhaustivo de la correlación entre los diagnósticos fenotípico y molecular de los pacientes con errores innatos de la inmunidad, confirmados en un hospital terciario de Cali, Colombia.

**Materiales y métodos.:**

Se desarrolló un estudio retrospectivo mediante la evaluación secuencial de las historias clínicas de los pacientes con errores innatos de la inmunidad que habían consultado al Servicio de Inmunología Clínica del Hospital Universitario del Valle.

**Resultados.:**

En el Servicio de Inmunología Clínica del Hospital Universitario del Valle se habían atendido 517 pacientes. Según la clasificación IUIS-2022, 92 pacientes (17,35 %) fueron diagnosticados con errores innatos de la inmunidad, de los cuales, 38 fueron sometidos a estudios genéticos. El grupo más prevalente de errores innatos de la inmunidad fue el de deficiencias predominantemente de anticuerpos (grupo III) (38/92 - 41.3 %). Se encontró un amplio espectro de defectos genéticos-nuevos y previamente reportados - incluyendo mutaciones en los genes *ATM, BTK, ERBIN, MAB21L2, RAG2, SAVI, SH2D1A, STAT1, SYK* y *TMEM173*. Entre los hallazgos menos frecuentes, se identificaron casos de síndrome de WHIM, aumento de la función del SYK y deficiencia de IL-7.

**Conclusiones.:**

El establecimiento del Servicio de Inmunología Clínica del Hospital Universitario del Valle ha emergido como un recurso fundamental para atender a personas con recursos financieros limitados en el suroccidente colombiano. El diagnóstico genético se logró en 38 casos (41.3 %) de pacientes con errores innatos de la inmunidad y cambió el diagnóstico en 24 pacientes (26 %).

Previously referred to as primary immunodeficiencies, these conditions are now understood as a diverse range of pathophysiology and are termed inborn errors of immunity. This group encompasses genetic diseases that affect various aspects of a patient’s immune system ^1^. Starting in the 20th century with the agammaglobulinemia of Bruton, a broad spectrum of diseases is being identified every year, with new gene mutations described, contributing to amplifying the phenotypic characteristics of each inborn error of immunity ^2^^,^^3^. Despite scientific advances, these immunological diseases are under-considered and have diagnostic delays in clinical practice. They do not receive accurate functional or genetic testing, subsequently resulting in complications with higher morbidity and mortality ^4^.

Innate errors of immunity are regulated by various legislations for orphan diseases. However, little progress has been made in these patients’ disease courses due to the high complexity of reaching a diagnosis ^5^. Therefore, the characterization of different variables of these pathologies, along with their classification within diagnostic subgroups, will be used for establishing public health measures and future research on these diseases ^6^.

The most recent update (2022) of the Phenotypical Classification for Human Inborn Errors of Immunity by the Expert Committee of the International Union of Immunological Societies (IUIS) identified 485 genetic defects responsible for these diseases ^7^. The recent broad access to genetic testing has facilitated the diagnosis of inborn errors of immunity based in their association with unique gene defects ^8^^,^^9^. Whole exorne sequencing, gene panels for immunodeficiency, and functional tests have improved performance and diagnosis, but they have high costs and limited access in emerging countries ^9^.

Since 2015, our group has been devoted to raising awareness, promoting early diagnosis, enhancing treatment, and ultimately improving the quality of life for patients with inborn errors of immunity. Over the years, we have published numerous case reports ^10^^-^^12^ and clinical research papers ^13^^-^^15^). Nevertheless, this marks our inaugural comprehensive paper, providing a holistic view of our insights into inborn errors of immunity in southwestern Colombia. We conducted an analysis based on patients’ medical records who had sought treatment at our university hospital and had received an inborn error of immunity diagnosis.

The objective of this study was to shed some light on the clinical, immune phenotype, and molecular diagnoses of patients with inborn errors of immunity attended in our clinical immunology service; also, to evaluate inborn errors of immunity diagnoses identified at a single center in Cali, Colombia. We assessed the access frequency of patients to genetic testing and how it influenced their diagnoses.

## Materials and methods

### Study design and population

We conducted a retrospective and descriptive study. We included children and adult patients who consulted between August 2015 and April 2022 to the Clinical Immunology Service of the *Hospital Universitario del Valle*, a highly complex institution in southwestern Colombia. The diagnosis of inborn errors of immunity was done according to the clinical and paraclinical criteria based on the European Society for Immunodeficiencies classification ^16^. In addition, the International Union of Immunology Societies (IUIS) classification of 2022 was used to determine the different phenotypic subgroups of inborn errors of immunity ^7^.

Patients included were selected conveniently through non-probabilistic sampling, sequentially evaluating all available medical records in the hospital information system with a diagnosis of inborn errors of immunity, from the most recent to the oldest.

We considered the approach successful when, throughout the follow-up from the initial evaluation to the final diagnosis, a patient was accurately classified into a specific subgroup and diagnosed with an inborn error of immunity.

### Molecular testing

In all the medical records, we looked for molecular tests. Reports evaluated included whole exorne sequencing, gene panels, Sanger sequencing for specific genes, fluorescence *in situ* hybridization, CytoScan™ arrays (Thermo Fisher Scientific), and others. Molecular testing was performed by insurance companies of the patients (Colombian healthcare system) through a kind donation from the Jeffrey Modell Foundation (207 genes panel from Invitae^®^) or whole genome sequencing donated by 3billion (Seul, Republic of Korea: https://3billion.io/).

Molecular studies were not conducted at the institution due to their unavailability, and functional tests were inaccessible due to difficulties with healthcare insurance.

### Statistical analysis

Descriptive statistical analysis was performed. Continuous variables were presented as mean and standard deviation or median and interquartile range, depending on the assumption of normality. Categorical variables were divided into proportions and compared using the chi-square test or Fisher’s exact test, as appropriate. A *priori* statistical significance level of a = 0.05 was established.

The correlation between quantitative variables was assessed with Pearson’s correlation coefficient and its respective coefficient of determination if at least one of the two variables met the assumption of normality. If not, the non-parametric Spearman’s correlation coefficient was employed. Analyses were performed with the statistical software RStudio™, importing the data from the Epi-Info database.

### Ethical considerations

The study was conducted according to the guidelines of the Declaration of Helsinki and approved by the ethics committee of the Hospital Universitario del Valle (May 6, 2022) in Cali, Colombia.

## Results

**Figure 1.**
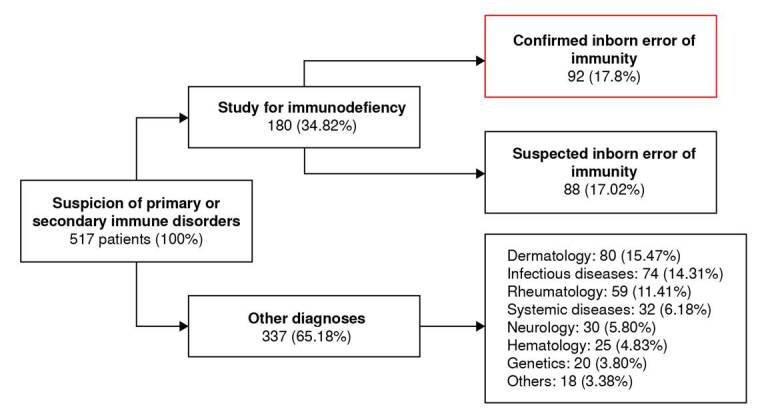
Flowchart of total patients. Subspecialized clinical immunology consultation experience. This chart details the number of patients evaluated in the immunology service, those considered to have immunodeficiency, and those diagnosed with an inborn error of immunity. A significant proportion of patients were also assessed for other diagnoses across various specialties, as shown in the distribution.

The Clinical Immunology Service at the Hospital Universitario del Valle evaluated 517 subjects (outpatient and inpatient), distributed as follows: 92 patients (17.8%) with a confirmed inborn error of immunity diagnosis, 88 (17.02%) were classified as high-suspicion for inborn errors of immunity or under study, 80 (15.47%) presented complex dermatological syndromes, 74 (14.31%) had chronic infectious diseases, 59 (11.41%) had rheumatological diseases, and 125 patients (24.17%) exhibited other clinical syndromes or immune manifestations (including systemic diseases and genetic syndromes) (figure 1 and supplement table 1).

Confirmed inborn errors of immunity cases involved 57 males (61.9%) and 25 females (38.1%). Children (< 18 years old) represented 68.47% (63/92) of the patients, even though the clinical immunology service was primarily established to cater to adult patients, constituting only 29 (31.53%). Furthermore, significant differences in age and classification subgroups at the time of final diagnosis were observed between those under 18 years old and those over 18 years old (p < 0.001). However, no significant differences were found when evaluating by gender across the different subgroups (p = 0.07).

The patients with confirmed inborn errors of immunity were primarily referred to the clinic due to severe, recurrent, or opportunistic infections. These referrals were aligned with the most classical warning signs for primary immunodeficiencies, as established by the Jeffrey Modell Foundation. However, some patients evaluated at the clinic also exhibited symptoms of autoinflammatory conditions and allergies, accounting for 24% of our patients with non-classical warning signs. Due to many cases occurring in childhood, we observed an average onset of symptoms at the age of two years (IQR = 1 -10), with a considerable delay between onset and the diagnosis of the inborn error of immunity, averaging seven years (IQR = 1-9 years).

Molecular tests were conducted in 38 patients, approximately half of the cases. Whole exorne sequencing was performed in 28 patients, and genetic panel testing was administered to 10 patients. We compared the significant differences between the initial phenotypic diagnosis and the final molecular diagnosis (table 1), and we found that 24 cases exhibited a significant change in diagnosis as a result of the molecular testing (table 1).

**Table 1.** Diagnoses by subgroups in patients with primary immunodeficiency disorders: A comparison of the initial phenotypic and final molecular diagnosis

Molecular diagnosis	Phenotypic diagnosis	Molecular test	Affected gene	Zigosity	Variant type	Sex	Age at diagnosis (years)
Subgroup 1							
IL-7 deficiency	Severe combined immunodeficiencvs Mendelian susceptibility to mycobacterial diseases	WES	IL-7	Horn	Path	M	19
Subgroup 2							
Noonan syndrome	Common variable immunodeficiency	WES	*PTPN11*, c.784C>T (P.L262F) *RAG2,* c.829dup (p.Y277Cfs*4)	Het	Path	M	13
Craniofrontonasal dysplasia	Hypogammaglobulinemia	WES	*EFNB1*, c.128+1G>C	Het	VUS	F	18
Netherton syndrome	Psoriasis	WES	*SPINK5*, c.1302+4A>T; c.2468dup (p.K824Efs*4)	Het	VUS	M	43
DiGeorge syndrome	Lymphopenia	Ins-Del	*22q11*			M	1
DiGeorge syndrome	Lymphopenia	Ins-Del	*22q11*			M	8
Ataxia telangiectasia	Hyper- IgM syndrome	Sanger	*ATM*, c.2921+1G>A	Horn	Path	M	12
Ataxia telangiectasia	Ataxia telangiectasia	NGS	*ATM*, c43delC (p.L15*); c.7884delT (p.l2629Yfs*2)	Compound Het	Path	M	4
Ataxia telangiectasia	Ataxia telangiectasia	WES	*ATM*, c.5557G>A (p.D1853N);	Het	VUS	F	1
Ataxia telangiectasia	Severe pneumonia	Sanger	c.7767delA (p.K2589Nfs*17)	Horn	Path	F	14
Hyper-lgE syndrome	Hyper-lgE syndrome	WES	No variants were detected			M	1
Hyper-lgE syndrome	Hyper-lgE syndrome	WES	*CR2* c.2728C>T (p.P913L) *JAK3* c.268G>A (p.V90M)	Compound Het	VUS	M	11
Hyper-lgE syndrome	Hyper-lgE syndrome	WES	*MAB21L2*, c.769 770delCT (p.L257Afs*126)	Het	Path	F	4
Hyper-lgE syndrome -AD due to STAT3	Hyper-lgE syndrome	WES	*STAT3* (p.N465S)	Het	Path	M	1
Hyper-lgE syndrome due to ERBIN	Hyper-lgE syndrome	WES	*ERBIN* c.2803G>A (p.D935N)	Het	VUS	M	3
Subgroup 3							
Common variable immunodeficiency	Common variable immunodeficiency	WES	No variants were detected			M	31
Common variable immunodeficiency	Common variable immunodeficiency	WES	No variants were detected			M	11
Common variable immunodeficiency	Common variable immunodeficiency	WES	No variants were detected			M	4
Common variable immunodeficiency	Common variable immunodeficiency	WES	*DNMT3B*	Het	VUS	F	6
Common variable immunodeficiency with molecular deficiency	Specific antibody deficiency and IgA deficiency	Panel for PID	*ATM*, c.1073A>G (p.N358S) *DLRE1C*, c.556G>C (P.V186L)	Compound Het	VUS	F	9
Common variable immunodeficiency	Common variable immunodeficiency	WES	No variants were detected			F	33
Selective IgA deficiency	Selective IgA deficiency	WES	No variants were detected			F	1
Selective IgA deficiency	Selective IgA deficiency	WES Trio	*IRF8*, c.1047C>T (p.C349=)	Het	VUS	M	0
SYK gain-of-function	Common variable immunodeficiency	WES	SYK	Het	Path	M	21
Agammaglobulinemia whitout genetic diagnosis	Vaccine-associated paralytic poliomyelitis	WES Trio	*AP3B1* and STXBP12	Het	VUS	F	0
RAG1 deficiency	Hypogamma IgG and IgA	WES Trio	*RAG1* c.2615T>G (p.L872*)	Het	Path	F	3
XLA (Bruton's agammaglobulinemia)	Agammaglobulinemia	Sanger	*BTK* c.557dupa (p.P187Afs*7)	Het	VUS	M	0
XLA (Bruton's agammaglobulinemia)	Agammaglobulinemia	Sanger	*B7K* c1385G>A (p.G462D)	Het	VUS	M	2
Activated PI3K delta syndrome	Hyper-IgM syndrome	WES	*PIK3R1* c.1425+1 G>T	Het	Path	M	14
Subgroup 4							
Autoimmune lymphoproliferative syndrome	Early autoimmune disorder	NGS	*MPO, ZNF341*, and TTC7A	Het	VUS	M	3
X-linked lymphoproliferative syndrome	Common variable immunodeficiency	WES	*SH2D1A*	Het	Path	M	36
Subgroup 6							
WHIM syndrome (warts, hypogammaglobulinemia, infections, and myelokathexis)	Congenital neutropenia	WES	*CXCR4*, c.10000T (p.R334*)	Het	Path	M	39
Mendelian susceptibility to mycobacterial diseases due to IL12(3 deficiency	Tuberculosis	WES	No variants were detected			F	18
STAT1 gain-of-function	Chronic mucocutaneous candidiasis	WES	*STAT1*, c.821 G>A (p.R274Q)	Het	Path	F	16
Subgroup 7							
STING-associated vasculopathy with onset in infancy	Autoinflammatory syndrome	WES	*TMEM173*	Het	VUS	F	18
Hyper-IgD syndrome	Autoinflammatory syndrome	WES Duo	*BRAT1*, c646C>T (p.Q216*)	Het	Path	M	6
Deficiency of IL-36 receptor antagonist	Pustular psoriasis	Sanger	*IL36RN,* c.200G>T (p.C67F)	Horn	Path	F	20
Subgroup 9							
RUNX1 loss-of-function	Hyper-IgE syndrome	WES	*RUNX1*	Het	Path	F	8

Het: Heterozygous; Horn: Homozygous; Path: Pathogenic; VUS: Variant of uncertain significance; WES: Whole exorne sequencing; Ins-Del: Insertion-deletion; PID: Primary immunodeficiencies

Some data may be missing due to an embargo, as these cases are currently undergoing further functional analysis.

A broad spectrum of genetic defects -novel and previously reported- were described, including mutations in the following genes: *ATM, BTK, ERBIN, MAB21L2, RAG2, SAVI, SH2D1A, STAT1, SYK*, and *TMEM173*. We highlight the cases of WHIM syndrome (warts, hypogammaglobulinemia, immunodeficiency, and myelokathexis), *SYTC* gain-of-function, *APDS2, SAVI*, and IL-7 deficiency. The variants in these genes are not described in the table as they are currently under embargo.

According to the 2022 IUIS classification, predominantly antibody deficiencies constituted the most frequent group of inborn errors of immunity in 36 patients (38/92 - 41.3%). Among them, common variable immunodeficiency was the prevailing diagnosis in nine patients (9/92 - 9.7%). All patients diagnosed with common variable immunodeficiency were older than four years, and the median B cell count was 354.6 cells/pl (IQR = 73 - 653.5). The common variable immunodeficiency was the most frequent phenotypic diagnosis that underwent significant changes following genetic testing in four cases, even leading to a reclassification in different subgroups.

A small proportion of patients were evaluated during hospitalization (8/92 - 8.69%), while 91.3% (84/92) did not require it, and follow-up was conducted on an outpatient basis. Throughout our follow-up, we have reported three deaths (two adults and one child) due to secondary causes to severe respiratory infections related to inborn errors of immunity.

## Discussion

A total of 517 patients were attended, and we focused on those 92 patients with a potential inborn error of immunity diagnosis to be confirmed. Remarkably, we achieved a molecular diagnosis in 38 cases (41.3%), a significant achievement for a middle-income country like Colombia ^17^. Nevertheless, we acknowledge the inherent limitations of a retrospective study, where patient evaluation and classification rely on medical records and laboratory tests authorized by the Colombian national healthcare system, making it challenging to ascertain the underlying genetic diagnosis with the phenotypical initial approach ^18^.

Initially, the outpatient clinic primarily catered to the adult population, but the unmet needs of the pediatric population, representing two-thirds of the patients, led to a higher proportion of adults being evaluated than reported In the literature ^19^^,^^20^. The primary focus was addressing innate errors of immunity in the adult population. Notably, early childhood subgroups, particularly those with more severe immunodeficiencies, were underrepresented due to this emphasis. The participation of an immunology clinic with expanded diagnostic tools may help balance the population groups being attended and reduce the number of patients without an etiological diagnosis ^21^.

A significant number of patients referred to the clinic presented with infectious, rheumatological, or allergic pathologies, among other conditions, that were either underdiagnosed or untreated. Clinical immunology services must implement comprehensive evaluations since many patients are referred by specialists but may not have an immunological condition ^22^. This integral assessment was an advantage of our service, as patients were attended by a physician with primary clinical expertise who was also an immunologist.

Given that the estimated incidence of severe combined immunodeficiency is 1 in 65,000 live births ^23^, our current diagnostic distribution shows underreporting of groups 1 and 2. Patients with severe combined immunodeficiency were managed in institutions with bone marrow transplantation capabilities, as this outpatient clinic did not handle such cases. Additionally, it is essential to note that Colombia does not conduct neonatal screening for immunodeficiencies, resulting in high mortality in their first months of life because the most severe phenotypes did not reach the clinical immunology outpatient clinic ^23^.

When evaluating the classification subgroups, the most prevalent subgroup according to the IUIS-2022 classification ^7^ was predominantly antibody deficiencies (42.9%), followed by combined immunodeficiencies with syndromic features (20.2%) and complement deficiencies (11.9%), consistent with previous international reports ^24^. In a multicentric study with data from 30 countries in Europe, Africa, and Asia, the prevailing subgroup was predominantly antibody deficiencies, accounting for 46.3% of cases ^25^. Additionally, reports from the *Grupo de Immunodeficiencias* Primarias at the *University of Antioquia* in Colombia observed a higher proportion of cases with predominantly antibody deficiencies (94%). However, only 10% of these patients underwent genetic studies ^26^.

The most frequent diagnoses were common variable immunodeficiency, selective IgA deficiency, IgG hypogammaglobulinemia, and hereditary angioedema type 1. These data are aligned with previous reports in Latin America ^24^ and Europe ^27^. However, compared with the genetic diagnosis, the common variable immunodeficiency was also the phenotypical diagnosis that changed the most. Due to the broad spectrum of inborn errors of immunity, many common variable immunodeficiency-like disorders could overlap, but are now distinguished with the new identification of gene mutations and disease reports ^28^. Thus, as seen in our patients with WHIM syndrome, SYKgaln-of-function, and combined immunodeficiency due to IL-7 deficiency, we strongly suggest undergoing genetic testing in all patients diagnosed with common variable immunodeficiency.

In inborn errors of immunity, even if patients develop symptoms at an early age, there is often a significant diagnostic delay. In our cohort, patients exhibited an average onset of symptoms at two years of age and experienced a diagnostic delay of seven years. This delay persists even for a phenotypical diagnosis, primarily due to limited access to genetic testing in Latin America ^29^^,^^30^. The role of an immunologist in these patients’ evaluation and follow-up helps to facilitate education and awareness processes, thereby enhancing the chances of diagnosing inborn errors of immunity and urging the healthcare system to prioritize further testing ^31^^,^^32^.

The mortality rate among patients with inborn errors of immunity mostly depends on the accuracy of their diagnosis ^33^. Depending on the subgroups, there is an increased risk of mortality due to complications such as infection, autoimmunity, or hematological malignancies ^34^. We observed a mortality rate of 3.2% (3/92) during our follow-up, consistent with data from other studies in which mortality in adults is lower in those who present with symptoms after six months of age ^35^. The variable mortality rate depends on the diagnosed inborn errors of immunity, emphasizing the significance of conducting adequate phenotype-genotype assessments for effective management ^36^^,^^37^.

A significant discrepancy exists between the initial clinical and phenotypical diagnoses and the subsequent molecular diagnosis, which could alter the treatment and prognosis for patients with inborn errors of immunity. The establishment of an immunology service enhances the diagnosis, care, and follow-up of patients with inborn errors of immunity. However, for a more comprehensive clinical approach to these patients, it is imperative to implement public policies that facilitate access to care, diagnostic studies, and treatment.

Due to the retrospective nature of our study, some data were missing because of incomplete clinical records. Genetic studies and functional tests were not performed in all patients with suspected inborn errors of immunity due to difficulties in accessing these services within Colombia’s healthcare system.

## Supplementary table

**Supplementary table 1.** Diagnoses by subgroups in patients with primary immunodeficiency disorders (N = 92)

Subgroup 1		n
	Hyper-IgM syndrome	1
	Severe combined immunodeficiency (SCID)	1
	Combined immunodeficiency due to IL-7 deficiency	1
Subgroup 2		
	Hyper-IgE syndrome without genetic confirmation	4
	Hyper-IgE syndrome due to STAT3	1
	Hyper-IgE syndrome due to ERBIN	1
	Ataxia telangiectasia	5
	Syndromic lymphopenia	2
	DiGeorge syndrome	1
	Severe antibody deficiency (SAD)	1
	Netherton syndrome and hyper-lgE syndrome	2
	Down syndrome with functional antibody deficiency	1
	Craniofrontonasal dysplasia	1
	Noonan combined immunodeficiency	1
Subgroup 3		
	Common variable immunodeficiency	11
	Selective IgA deficiency	9
	Hypogammaglobulinemia IgG	7
	Hypogammaglobulinemia IgG and IgA	3
	Agammaglobulinemia	3
	SYK gain-of-function	1
	RAG1	1
	Bruton's agammaglobulinemia	1
	APDS2 (Activated PI3K Delta syndrome 2)	1
	Combined immunodeficiency	1
Subgroup 4		
	ALPS (Autoimmune lymphoproliferative syndrome)	1
	X-linked lymphoproliferative syndrome	1
	Immuno-dysregulation syndrome	1
Subgroup 5		
	Benign cyclic neutropenia	3
	Leukocyte adhesion deficiency	1
Subgroup 6		
	WHIM syndrome (Warts, hypogammaglobulinemia, infections, myelokathexis)	1
	Innate immunity deficiency	1
	STAT1 gain-of-function mutation	1
	Mendelian susceptibility to mycobacterial diseases due to IL-12β deficiency	1
Subgroup 7		7
	Autoinflammatory syndrome	4
	Autoinflammatory hyper-IgG syndrome	2
	STING-associated vasculopathy with onset in infancy (SAVI)	1
	Deficiency of IL-36 receptor antagonist (DURA)	1
Subgroup 8		
	Hereditary angioedema type 1	7
	Hereditary angioedema to be classified	1
Subgroup 9		
	RUNX1	1
	Gene-related immunodeficiency and medullary failure due to an heterozygous mutation	1
	Medullary insufficiency syndrome	1
Subgroup 10		
	Adult-onset immunodeficiency with susceptibility to mycobacteria	1
